# Imaging using radiolabelled targeted proteins: radioimmunodetection and beyond

**DOI:** 10.1186/s41181-020-00094-w

**Published:** 2020-06-23

**Authors:** Javad Garousi, Anna Orlova, Fredrik Y. Frejd, Vladimir Tolmachev

**Affiliations:** 1 Department of Immunology, Genetics and Pathology, Uppsala University, Uppsala, Sweden; 2 Department of Medicinal Chemistry, Uppsala University, Uppsala, Sweden; 3 Science for Life Laboratory, Uppsala University, Uppsala, Sweden; 4 Research Centrum for Oncotheranostics, Research School of Chemistry and Applied Biomedical Sciences, Tomsk Polytechnic University, Tomsk, Russia

**Keywords:** radionuclide, imaging, antibodies, antibody fragments, scaffold proteins

## Abstract

The use of radiolabelled antibodies was proposed in 1970s for staging of malignant tumours. Intensive research established chemistry for radiolabelling of proteins and understanding of factors determining biodistribution and targeting properties. The use of radioimmunodetection for staging of cancer was not established as common practice due to approval and widespread use of [^18^F]-FDG, which provided a more general diagnostic use than antibodies or their fragments. Expanded application of antibody-based therapeutics renewed the interest in radiolabelled antibodies. RadioimmunoPET emerged as a powerful tool for evaluation of pharmacokinetics of and target engagement by biotherapeutics. In addition to monoclonal antibodies, new radiolabelled engineered proteins have recently appeared, offering high-contrast imaging of expression of therapeutic molecular targets in tumours shortly after injection. This creates preconditions for noninvasive determination of a target expression level and stratification of patients for targeted therapies. Radiolabelled proteins hold great promise to play an important role in development and implementation of personalised targeted treatment of malignant tumours. This article provides an overview of biodistribution and tumour-seeking features of major classes of targeting proteins currently utilized for molecular imaging. Such information might be useful for researchers entering the field of the protein-based radionuclide molecular imaging.

## Brief historical overview

**Fig. 1:**
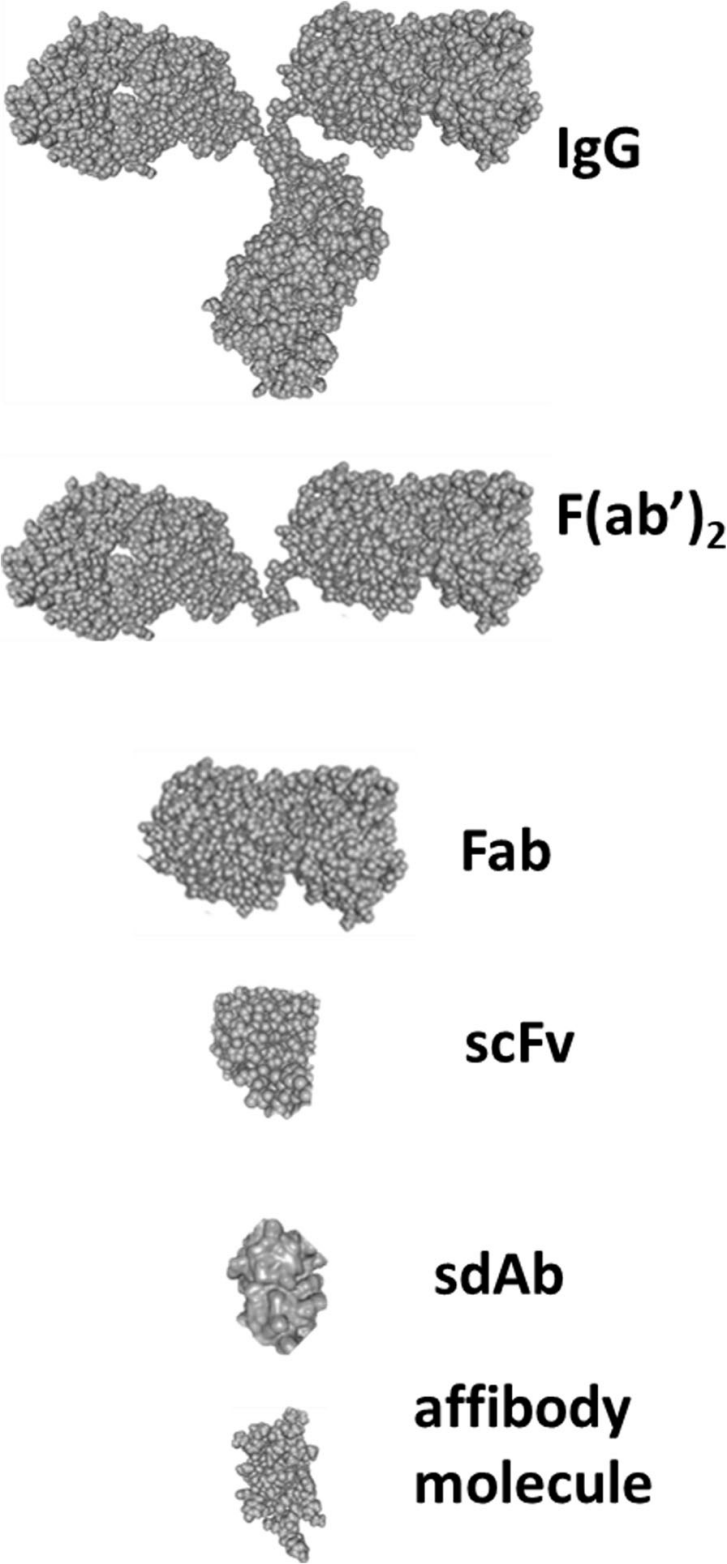
Relative size of proteins applied for radioimmunodetection and molecular imaging. Images are taken from Protein Data Bank ( https://www.rcsb.org/)

The first attempt for radionuclide imaging of tumours using radiolabelled antibodies was made in the seventies to avoid shortcomings of existing methods for cancer staging. ^131^I-labelled polyclonal antibodies against carcinoembryonic antigen (CEA) were the first probes used in radioimmunodetection (RID) (Goldenberg et al. 1974; Mach et al. 1974). A great boost to this approach was given by the invention of the hybridoma technology (Kohler and Milstein, 1975) permitting production of uniform monoclonal antibodies (mAbs) with defined specificity and affinity to tumour-associated antigens. During the following twenty-five years, intensive research identified biologic barriers for efficient tumour targeting, such as poor perfusion, poor extravasation rate and slow diffusion in extracellular space (Jain 1990). Some solutions for such problems, such as reduction of size by the use of Fab and (Fab)_2_ fragments have been identified (Delaloye et al. 1986). By the mid-nineties, RID enabled sensitivity of more than 70% and specificity of 80%, even in otherwise occult tumours (Bischof Delaloye and Delaloye 1995). However, regulatory approval of [^18^F]-FDG and explosive growth of positron emission tomography (PET) installations number was fatal for RID-based tumour staging. One of the pioneers in RID, Prof. Angelica Bischof Delaloye wrote in 2000: “The more and more generalized availability of positron emission tomography (PET) with Fluorine-18 fluorodeoxyglucose (FDG) for diagnosis and staging of malignant diseases will probably definitively seal the fate of radioimmunodiagnosis as it has been conceived up until now.“ (Bischof 2000). This prophecy was absolutely correct. Interestingly, Prof. Bischof Delaloye predicted in the same review the contemporary direction of RID: “Radiolabeled antibodies will probably no longer be used for lesion detection, which is more reliably made with FDG-PET, but for lesion characterization. The more lesions we detect, the more we need to know their nature to base patient management on reliable data.” Indeed, targeting is one of the most promising approaches to treatment of disseminated cancer. The level of expression of a therapeutic target is often a critical predictive biomarker for antibodies and antibody-drug conjugates therapeutic efficacy. Thus, a sufficiently high accumulation of radiolabelled analogues of therapeutic antibodies in metastases should be a predictor that the patient would benefit from targeted therapy. Recognition of this gave a second wind to RID. Moreover, continuing development of biotechnology has enabled the creation of engineered antibody formats offering pharmacokinetics features more suitable for imaging than features of intact IgG monoclonal antibodies. Furthermore, novel non-immunoglobulin-based high-affinity protein binders have been invented, which could be utilized as imaging probes (Bedford et al. 2017; Krasniqi et al. 2017). Currently, several types of proteinaceous imaging probes with different characteristics and pharmacokinetics are available (Fig. 1). Some of their important features, which are essential for molecular imaging, are briefly overviewed below.

### Intact monoclonal antibodies

Therapeutic mAbs specific to receptor tyrosine kinases (RTK), such as human epidermal growth factor receptor (EGFR) or human epidermal growth factor receptor type 2 (HER2) have been used for targeted treatment of different types of cancers since 90s. Labelling of these mAbs with radionuclides enabled visualization of their therapeutic targets in clinical trials (Behr et al. 2001; Dijkers et al. 2010; Even et al., 2017). Long residence time of mAbs in the circulation necessitates the use of relatively long-lived radionuclides. For SPECT applications, labelling of intact IgG was performed mainly using ^111^In (T_1/2_= 2.8 d) (Behr et al. 2001; Perik et al. 2006; Desar et al. 2010). Since better sensitivity and resolution of PET compared to SPECT might improve imaging using RID, labelling of mAbs with long-lived positron emitters, such as ^89^Zr, ^64^Cu, ^124^I and ^86^Y, is gaining an increasing interest (van Dongen et al. 2007; Lamberts et al. 2015; Wei et al. 2018 ). Owing to a dedicated work of Prof. van Dongen and his team from Vrije Universiteit Amsterdam, GMP-produced long lived positron emitters ^124^I (T_1/2_= 4.17 d) and ^89^Zr (T_1/2_= 3.27 d) are currently commercially available and protocols for their coupling to mAbs are established (Verel et al. 2003; Verel et al. 2004). These nuclides are, probably, the optimal ones for labelling of intact IgG, as typically several days between injection and imaging using these bulky proteins are required to obtain reasonable contrast.

It would not be an exaggeration to state that immunoPET is being established as a valuable tool in drug development coupled with a steady and rapid growth in development of antibody-based therapeutics. Currently, over 570 antibody therapeutics are at various clinical trial phases, including 62 in late-stage clinical studies (Kaplon and Reichert 2019). Quantitative non-invasive PET imaging may be used from preclinical to clinical stages providing important information concerning pharmacokinetics of mAbs and their derivatives, particularly the extent of their accumulation in tumours (Lamberts et al. 2015). The use of quantitative imaging might also be helpful in finding of an optimal antibody dose for therapy. A search in a clinical trials data base (https://clinicaltrials.gov/ ) results in dozens of hits concerning development of ^89^Zr-labelled mAbs for support of clinical trials.

Still, the future of intact IgG as tools for patients’ stratification is unclear. There are apparent advantages of the use of radiolabelled mAbs as companion diagnostics:

- Radiolabelled counterpart reflects not only the level of expression of a target in tumours, but also its accessibility (Fleuren et al. 2011; Terwisscha van Scheltinga et al. 2017):
- Safety of therapeutic mAbs is well known, which reduces the number of necessary tests for their imaging tracer derivatives;
- Labelling with ^89^Zr is well-established and straightforward. While there are some indications of limited in vivo stability of a complex of ^89^Zr with the most commonly used chelator, DFO, there is an active development of new chelators providing more stable complex with ^89^Zr (Heskamp et al. 2017). Particularly promising are DFO* and its derivatives providing high stability of ^89^Zr label in vivo (Vugts et al. 2017; Raavé et al. 2019).

However, there are also apparent issues associated mainly with the size of IgG:

- Slow clearance of mAbs from blood, which causes a low signal-to-background ratio in imaging and reduces sensitivity. Clinical studies indicated that the best imaging contrast could be obtained only 4-7 days after injection (Dijkers et al. 2010; Even et al., 2016);
- A noticeably high dose burden to patients due to the use of long-lived positron emitters is of concern (Gaykema et al. 2013). Indeed, an effective dose for ^89^Zr-labelled mAbs is estimated to be in the range from 0.38 to 0.61 mSv/MBq and the red marrow dose might be as high as 0.69 mSv/MBq (Pandit-Taskar et al. 2014; Börjesson et al. 2009; Makris et al. 2015; Laforest et al. 2016; O'Donoghue et al. 2018; Ulaner et al. 2018).
- Slow extravasation and diffusion rates of IgG in tumour interstitium (Jain 1999; Schmidt and Wittrup 2009) ;
- Unspecific accumulation in tumours due to tumour enhanced permeability and retention (EPR) effect typical for large macromolecules (Maeda et al. 2000). Tumour uptake of unspecific antibodies might be up to 50% of specific ones (Lub-de Hooge et al. 2004; Terwisscha van Scheltinga et al. 2017). This is associated with a risk of false-positive diagnoses (Ulaner et al. 2016).

Overall, imaging using radiolabelled intact immunoglobulins seems to be more suitable for studies of pharmacokinetics and target engaging of new antibody-based therapeutics than for stratification of patients for targeted therapies. It is important to mention, however, that biodistribution of radiolabelled mAbs does not reflect exactly the situation in a therapeutic approach with cold antibodies. The amount of antibodies and time duration of injection/infusion is in most cases completely different. Both these parameters influences of course the distribution and the penetration of the antibodies into the tumour.

### Fab and (Fab’)_2_ fragments

The major issues in the use of intact IgG for radionuclide imaging are associated with their large size. Digestion of antibodies with the enzymes pepsin or papain results in the smaller fragments Fab (ca. 55 kDa) and (Fab’)_2_ (ca. 110 kDa) (Fig. 1). These fragments preserve the capacity of the parental IgG to bind specifically and selectively to their molecular targets. Both types of fragments have been radiolabelled and extensively evaluated for imaging. Multiple studies confirmed that these fragments clear much faster from blood than parental mAbs, often providing appreciably better contrasts (Divgi and Larson 1989; Goldenberg et al. 1990; Freise and Wu 2015). A good contrast might be obtained on the day of injection or the day after injection, which enables the use of relatively short-lived radionuclides, such as ^99m^Tc (T_1/2_= 6 h), or medium half-life positron emitters ^55^Co (T_1/2_= 17.5 h), ^64^Cu(T_1/2_= 12.7 h), ^76^Br (T_1/2_= 16.2 h), and ^86^Y(T_1/2_= 14.7 h). Methodology for production of medium half-live positron emitters using low-energy cyclotrons is well developed (see e.g. Aluicio-Sarduy et al. 2018; Ellison et al. 2020; Mastren et al. 2015; Valdovinos et al. 2017). Their use would be associated with a noticeable reduction of absorbed dose to patients compared with the use of ^89^Zr. Unfortunately, production of these nuclides (except from ^64^Cu) is established only in a few PET centres, which makes their translation into clinical practice slow.

The ^99m^Tc-labeled anti-EpCAM murine Fab fragment nofetumomab merpentan (NR-LU-10, Verluma) has been approved for staging of breast, lung, gastrointestinal, ovary, bladder, kidney, cervix, and pancreas carcinomas and has been found useful. Imaging using [^99m^Tc]Tc-NR-LU-10 demonstrated reasonable sensitivity (up to 90% for primary tumours) in imaging of lung cancer (Breitz et al. 1997; Vansant et al. 1992). However, there was a noticeable fraction (24%) of false-positive findings (Rusch et al. 1993). Another antibody fragment from this time is the ^99m^Tc-labeled anti-CEA Fab fragment arcitumomab (CEA-scan) derived by proteolytic pepsin cleavage from the parental murine antibody IMMU-4, and approved for diagnostic imaging of colorectal cancers (Behr et al. 1995; Moffat Jr et al. 1996). Lately, an efficient production of antibody fragments in non-mammalian cells has been established (for a review see, e.g., Spadiut et al. 2014).

Summarising, the advantage of the use of radiolabelled Fab and (Fab’)_2_ fragment of therapeutic mAbs are:

- Shortening the time between injection and imaging;
- Often, better contrast in comparison with intact IgG;
- Lower absorbed dose to patients.
- Rapid transformation of therapeutic IgG to an imaging fragment by proteolytic digestion for initial preclinical assessment.

Still, there are some issues as:

- Decrease of apparent binding affinity compared to IgG. Particularly, this is typical for Fab fragments due to the loss of the avidity effect of bivalent binding;
- Both Fab and (Fab)_2_ are still too large to have efficient extravasation;
- Both Fab and (Fab)_2_ are still above the EPR border (45 kDa for globular proteins (Wester and Kessler 2005)).

### Engineered antibody fragments

Further progress in development of protein-based imaging probes was associated with the development of single chain variable fragments (scFv). scFvs have a molecular weight of ~25 kDa, i.e. they are smaller than Fab. scFvs have only one binding site, i.e. they are monovalent, but scFv’s with subnanomolar affinities to their targets have been generated (Schier et al. 1996). In preclinical models, radiolabelled scFv’s demonstrated good imaging contrast, however, the absolute tumour uptake was quite low (below 2 %ID/g) [Adams et al. 1993; Adams et al. 1998]. Mathematic modelling suggest that that the reason for such low accumulation is a suboptimal ratio between the blood clearance and extravasation rates (Thurber et al. 2007). Engineering of the dimeric bivalent form of a scFv (diabody, ~50 kDa) and/or fusion of scFv to the Fc region of IgG (minibody, ~80 kDa and scFV-Fc, ~105 kDa) enabled to obtain higher accumulation in the tumours (Olafsen and Wu 2010). An apparent price was that the EPR limit was exceeded by such constructs. An unspecific uptake in target-negative control xenograft tumours compared to target-positive was 9-16% for ^124^I-labeled anti CA19-9 diabody (Girgis et al. 2011), 16% for anti-CEA diabody ^18^F-T84.66 (Cai et al. 2007), 27-32% for anti-PSCA diabodies [^124^I]-A2cDb and [^124^I]-A2cDb-800 (Zettlitz et al. 2018) and 20% for ^89^Zr-labelled diabody construct derived from anti-PSMA antibody (Viola-Villegas et al. 2014) at optimal imaging time. Thus, these interesting formats permit good imaging contrast within 24 h after injection, but do not eliminate risks of false-positive findings due to unspecific accumulation.

Probably the best approach for development of immunoglobulin-based imaging probes is the use of single domain antibodies (sdAb), derived from either phage display human antibody repertoires (designated as VH or dAbs) or from camelid immunoglobulins (designated VHH or nanobodies) (Vaneycken et al. 2010). The molecular weight of sdAb is in the ranges of 12-15 kDa, i.e. they are the smallest antibody fragments capable of specific binding to antigens. Camelid antibodies comprise both normal IgG’s and such ones that only one heavy chain. This allows the generation of an imaging probe based on the variable heavy chain domain only, with a molecular weight of only 15 kDa. This provides better relation between extravasation and excretion rates for sdAb than for scFv (Thurber et al. 2007) and permits high contrast imaging at the day of injection. A recent study in mice confirmed that the monomeric form of sdAb extravasate rapidly (much rapider than IgG) and distributed uniformly inside tumour (Debie et al.; 2020). Rapid clearance of nanobodies enabled labelling using ^99m^Tc or short-lived positron-emitting nuclides, such as ^68^Ga (T_1/2_ = 67.7 min) (Xavier et al. 2013; Bala et al., 2018) and ^18^F (T1/2 = 109.7 min) (Xavier et al. 2016; Vaidyanathan et al. 2016; Bala et al. 2016, Bala et al., 2019), reducing absorbed doses. Nanobodies binding to molecular targets such as epidermal growth factor (EGFR) (Gainkam et al. 2008; Huang et al. 2008), human epidermal growth factor type 2 (HER2) (Xavier et al. 2013; Vaidyanathan et al. 2016; D'Huyvetter et al. 2017; Xavier et al. 2016), CD20 (Krasniqi et al. 2017), carcinoembryonic antigen (CEA) (Vaneycken et al. 2010), prostate-specific membrane antigen (PSMA) (Evazalipour et al. 2014; Chatalic et al. 2015), macrophage mannose receptor (MMR) (Blykers et al. 2015; Movahedi et al. 2012) and programmed death-ligand 1 (PD-L1) (Broos et al. 2017) have been evaluated as imaging probes. Clinical studies confirmed that ^68^Ga-labelled anti-HER2 VHH provided very good imaging in patients (Keyaerts et al. 2016).

Advantages of sdAb as imaging probes include

- Size in the range permitting both efficient extravasation and clearance of an unbound tracer from circulation;
- Small size of imaging probe preventing unspecific accumulation in tumours due to the EPR effect;
- Efficient selection via immunisation of camelidae permitting a rapid selection of binders with a single digit nanomolar affinity

Site-specific incorporation of chelators in nanobodies is possible using a Sortase A-mediated ligation (Massa et al. 2016) enabling generation of a homogenous labelled tracer. Alternatively, a site-specific coupling of ^99m^Tc(CO)_3_ to histidine tags of nanobodies is possible (Waibel et al. 1999). This methodology has been applied for labelling of nanobodies targeting, for example, EGFR (Gainkam et al. 2008), CEA (Vaneycken et al. 2010), and PD-L1 (Broos et al. 2017). A common issue for nanobodies is a high renal reabsorption, a feature common for many other small proteins (see next chapter).

### Non-immunoglobulin engineered scaffold proteins

The Nobel Prize-awarded invention of phage display (Smith 1985) has opened the way to selection of peptide binders to a variety of molecular targets. However, generation of binders with sufficient affinity for in vivo molecular imaging became possible only after introduction of so called scaffold proteins (Nygren and Skerra 2004; Binz et al. 2005; Skerra 2007). Such proteins contain a robust framework (scaffold) providing structural stability and rigidity. Some amino acids on the scaffold surface are randomized to develop a combinatorial library. The use of molecular display techniques permits selection of binders with desirable specificity and selectivity. The presence of scaffold reduces an entropy penalty and enables selection of binders with very high affinity (down to low picomolar range).

Currently, several types of promising scaffold proteins are under evaluation for the use as imaging agents, e.g., Affibody molecules, affilins, avimers, affitins, Obodies, fynomers, DARPins, cysteine knots, anticalins and adnectins (Vazquez-Lombardi et al. 2015; Simeon and Chen 2018).

Engineered scaffold proteins (ESP) are quite small with a typical molecular weight in the range between 4 and 20 kDa, which meets the requirements for an optimal balance between extravasation and excretion rates (Schmidt et al., 2009). Their affinity is sufficiently high for imaging applications. Therefore, a number of scaffold proteins were evaluated for radionuclide imaging (for review see (Miao et al. 2011; Stern et al. 2013; Krasniqi et al. 2018)). The small size of ESP excludes the influence of EPR on the tumour uptake making the imaging highly specific. It has to be noted that nearly all ESPs undergo an efficient reabsorption in proximal tubuli of kidneys after glomerular filtration. When radiometal labels are used, the radionuclides are retained in kidneys. Despite this, clinical studies demonstrated that imaging of metastases in the lumbar area is possible using ^111^In- and ^68^Ga-labeled affibody molecules (Sörensen et al. 2014; Sörensen et al. 2016). Moreover, imaging of an adrenal metastases was documented using [^111^In]In-ABY-025 affibody molecule (Sörensen et al. 2014).

The short overview of ESPs evaluated for in vivo imaging is provided below.

#### Affibody molecules

The Affibody scaffold consists of three tightly packed alpha-helices, stabilized by a hydrophobic core. The size is only 58 amino acids and the molecular weight of affibody molecules is thus 6-7 kDa making them one of the smaller scaffolds available (Frejd and Kim 2017; Ståhl et al. 2017). Thirteen amino acids in helices 1 and 2 are randomized to create a combinatorial library. Phage or staphylococcal display enables selection of affibody binders with affinities from a few nanomolar to a few picomolar K_D_. Several affibody molecules with subnanomolar affinity binding with high affinity to HER2 (Orlova et al., 2006; Ahlgren et al. 2010; Kramer-Marek et al. 2011), HER3 (Orlova et al. 2014; Da Pieve et al. 2016), IGF-1R (Orlova et al. 2013), EGFR (Tolmachev et al. 2009a; Tolmachev et al. 2010a), CAIX (Honarvar et al. 2015; Garousi et al. 2016a), PDGFRβ (Tolmachev et al. 2014; Strand et al. 2014) and VEGFR2 (Mitran et al., 2018) have demonstrated an apparent potential as probes for radionuclide molecular imaging in preclinical settings. Clinical evaluation demonstrated that anti-HER2 affibody ABY-025 provides a high contrast specific imaging of HER2 and has a good dosimetry when labelled with ^111^In (Sörensen et al. 2014) and ^68^Ga (Sörensen et al. 2016; Sandström et al. 2016; Sandberg et al. 2017). An important feature of affibody molecules is a rapid re-folding in physiologic conditions after denaturing (Arora et al. 2014). This permits the use of elevated temperatures (up to 95 ^o^C), pH in the range from 3.5 to 11.5 and lipophilic solvents during labelling and purification.

Targeting properties of ^124^I-labeled anti-HER2 ZHER2:342 affibody molecule and anti-HER2 therapeutic monoclonal antibody trastuzumab were compared in mice bearing gastric cancer NCI-N87 xenografts with high HER2 expression (Orlova et al. 2009) (Fig. 3). [^124^I]I-ZHER2:342 provided tumour-to-blood ratio of 8±2 already at 6 h after injection while the highest tumour-to-blood ratio of 1.3±0.4 was reached by [^124^I]I-trastuzumab only 72 h after injection. Targeting properties of radiometal (^111^In)-labelled anti-HER2 affibody molecules ABY-025 was compared with the properties of [^111^In]In-CHX-A”-trastuzumab in prostate cancer DU-145 xenografts with low HER2 expression (Malmberg et al. 2011). The tumour-to-blood ratios for [^111^In]In-ABY-025 were 27±6 and 47±13 at 2 and 6 h, respectively. The best tumour-to-blood ratio for [^111^In]In-CHX-A”-trastuzumab, 18±7, was obtained only at 72 h after injection. These studies clearly demonstrated the advantage of affibody molecules over antibodies as imaging probes. Unfortunately, such head-to-head comparisons are not frequently published. For the moment, affibody molecules are probably the most studied type of ESP-based probes for radionuclide molecular imaging.

Besides small size and robustness, affibody molecules offer following advantages as potential imaging probes:

- Affibody molecules are amenable for peptides synthesis. This enables site-specific incorporation of a variety of chelators and prosthetic groups for radiolabelling in any desirable position (Orlova et al. 2010; Perols et al. 2012; Rosik et al. 2014);
- Introduction of a unique cysteine into the cysteine-free affibody scaffold permits site-specific labelling of recombinantly-produced affibody molecules using maleimido-mediated thiol-directed chemistry. This might be used for coupling of different chelators for radiometals (Tolmachev et al. 2008; Tolmachev et al. 2012; Altai et al. 2013) or prosthetic groups for radiohalogens (Namavari et al. 2008; Tolmachev et al. 2009b; Kramer-Marek et al. 2008; Su et al. 2014);
- Placement of cysteine at C-terminus provides N_3_S chelators (formed by thiol group of cysteine and amide nitrogens from adjacent amino acids) for site-specific labelling with ^99m^Tc, ^188^Re or ^186^Re (Altai et al. 2012; Altai et al. 2014; Oroujeni et al. 2018a, 2018b).
- Histidine-containing tags might be used for site-specific labelling using [^99m^Tc]Tc(CO)_3_ (Tolmachev et al. 2010b; Orlova et al. 2013; Orlova et al. 2014; ). Modification of position and composition of such tags enables optimisation of biodistribution of radiolabelled affibody molecules (Hofström et al. 2013).

Importantly, the site-specific labelling provides homogenous well-defined conjugates with reproducible biodistribution properties. Altogether, this enables selection of an optimal imaging strategy (see below).

#### ADAPTs

ADAPTs (ABD-Derived Affinity Proteins) have been developed using a 46-amino acid 5 kDa minimized scaffold derived from an engineered albumin-binding domain (ABD) (Garousi et al. 2015). The ABD folds spontaneously in a three-helix structure, and the folding is independent of disulphide bridges. An important feature of ABD is its capability to refold after chemical or thermal denaturing (Nilvebrant and Hober 2013 a). A team led by Prof. Hober (KTH-Royal Institute of Technology, Stockholm) created a library enabling selection of binders to different targets while high-affinity binding to albumin was preserved. ABD variants binding to TNFα, HER3 and HER2 were selected using this library (Nilvebrant et al. 2014, Nilvebrant et al. 2013 b; Nilvebrant et al. 2011). These novel binding variants were designated as ADAPTs (Nilvebrant et al. 2014). To provide rapid blood clearance, which is desirable for imaging probes, binding to albumin was completely eradicated for anti-HER2 variants. A variant ADAPT6 was selected with an affinity to HER2 of about 1 nM and no measurable binding to albumin (Garousi et al. 2015). During studies in immunocompromised mice bearing human ovarian carcinoma SKOV-3 with high HER2 expression, ADAPT6 provided 1 h after injection a tumour-to-blood ratio of 43 ± 11 with an ^111^In-label and 17.5 ± 0.3 with ^68^Ga (Garousi et al. 2015). The tracer provided a clear discrimination between xenografts with high and low HER2 expression. In further studies, the molecular design of the ADAPT6-based probe was refined. Influence of composition of histidine-containing tags (Lindbo et al. 2016), composition of N-terminus (Garousi et al. 2016b) and C-terminus (Garousi et al. 2017; Lindbo et al. 2018a) sequences, position of labels (Lindbo et al. 2018a), and chemical nature of a radionuclide (Lindbo et al. 2018a) on biodistribution and targeting properties of ADAPT6 were evaluated. Radionuclides such as ^111^In, ^68^Ga, ^125^I (as a surrogate for ^123^I or ^124^I) and ^99m^Tc were evaluated as labels for ADAPTs. As a result of these studies, an optimized variant of ADAPT6 provided a substantial increase of tumour-to-liver and tumour-to-bone ratios (Lindbo et al. 2018a). This is essential due to the frequent occurrence of liver and bone metastases from many cancers.

Interestingly, monomeric form of ADAPT6 provides more efficient tumour targeting than dimeric forms despite appreciably higher affinities of dimers (Garousi et al., 2019a). This finding corroborates with data for monomeric and dimeric affibody molecules (Tolmachev et al. 2009b; Cheng et al. 2008 ) and is a strong indication that small size is very important for efficient tumour imaging using ESP.

A preliminary report from a clinical study including 10 primary breast cancer patients shows that i.v. administration of [^99m^Tc]Tc-ADAP6 is safe and do not cause any adverse effects (Tolmachev et al. 2019) and is efficacious, as primary tumours could be visualized in all ten patients.

Overall, emerging data suggest that ADAPTs have the same advantages and disadvantages as affibody molecules for the use as imaging probes.

#### DARPins

The DARPin (designed ankyrin repeat protein) scaffold consists of 4-6 repeating 33-amino acids blocks, each organized as a β-turn and two antiparallel α-helices (Plückthun 2015; Boersma 2018). The molecular weight of DARPins is 14-18 kDa, depending on the number of repeats. The amino acids in both helices and loops can be randomized to create a selection library. DARPin binders with picomolar affinity can be selected to different targets (Zahnd et al. 2010; Stefan et al. 2011). Autoradiography study demonstrated good DARPins penetration in the tumor mass (Zahnd et al. 2010). The development of biomedical applications of DARPins is mainly focused on therapy. For radionuclide imaging, only two anti-HER2 DARPIns, G3 (Goldstein et al. 2015) and 9_29 (Vorobyeva et al. 2018), have been evaluated. DARPin G3 has a molecular weight of 14.3 kDa and binds to HER2 with the affinity of 91 pM. Molecular weight of 9_29 is 18.2 kDa, its binding affinity to HER2 is 3.8 nM. Recent direct comparison of ^125^I- and ^99m^Tc-labeled DARPins demonstrated that G3 is a preferable DARPin format providing much better imaging contrast compared with 9_29 with both ^125^I- and ^99m^Tc-labels (Deyev et al. 2019). Radioiodine label provided the best imaging contrast. For example, the tumour-to-blood ratio of 94±47 was obtained 6 h after injection. Further study demonstrated that a direct, Chloramine-T-mediated radioiodination provides a probe with better imaging properties than the same DARPin site-specifically radioiodinated using iodo-((4-hydroxyphenyl)ethyl) maleimide due to lower hepatobiliary excretion of directly iodinated variant (Vorobyeva et al. 2019).

#### Knottins

A scaffold of knottins (other designation cystine knot peptides) is formed by three anti-parallel β-strands linked by loops and three disulphide bonds (Colgrave and Craik 2004; Kintzing and Cochran 2016). A typical knottin consists of 30 amino acids and has a molecular weight of ca. 4 kDa, which makes them the smallest developed scaffold proteins. An important feature of knottins is their thermic and chemical stability, which is essential for selection of labelling strategy. Knottins can be efficiently produced by peptide synthesis (Avrutina 2016) and recombinantly in bacterial hosts (Schmoldt et al. 2005), which makes their manufacturing relatively cheap. A number of different knottin variants targeting integrins in neovasculature were labelled with ^18^F (Jiang et al. 2013; Jiang et al. 2014), ^64^Cu (Jiang et al. 2010) and ^111^In (Jiang et al. 2012) and evaluated for in vivo radionuclide imaging. Radiolabelled knottins demonstrated tumour-to-blood ratios of more than five within two hours after injection. Interestingly, the renal reabsorption of radiometal-labelled knottins was several-fold lower compared to other ESP, which probably indicates lower affinity of this scaffold to scavenger receptors in proximal tubuli of kidneys.

#### Adnectins

Adnectins were developed using the scaffold of the tenth domain of fibronectin type III (10Fn3) (Lipovsek 2011). This 94-amino-acid (ca. 10 kDa) cysteine-free scaffold is arranged as a β-sandwich containing seven strands and six connecting loops. Selection of high affinity adnectin binders is possible using phage, mRNA and yeast-surfcace display (Koide et al. 2012). Adnectin-based targeting was used mainly for development of therapeutics (Simeon and Chen 2018). For radionuclide imaging, the ^64^Cu-labeled FN3(CD20), binding to lymphoma-associated CD20 with affinity of 20 nM was evaluated (Natarajan et al. 2013). This tracer demonstrated specific accumulation in CD20-expressing Ramos xenografts in mice and a tumour to blood ratio of 4 was obtained 24 h after injection.

[^18^F]F-BMS-986192 adnectin was evaluated for the imaging of the programmed death protein ligand (PD-L1) for measuring this target expression in tumours for stratification of patients for immunotherapy (Donnelly et al. 2018). The labelled adnectin has affinity to PD-L1 of 35 nM. A specific imaging of PD-L1-positive xenografts in mice was demonstrated 2 h after administration of [^18^F]F-BMS-986192 with the tumour-to-blood ratio of approximately 2.

## Influence of labelling chemistry on imaging

It is important to understand the role of radiolabelling chemistry for adequate interpretation of biodistribution and imaging data. Several reviews have been published on this topic (Tolmachev and Orlova 2010; Tolmachev and Stone Elander 2010), and this section provides only a brief summary, focussing on two aspects of labelling chemistry, residualizing properties of labels and the influences of labels on biodistribution of imaging probes.

### Residualizing vs non-residualizing labels

Binding of an imaging probe to a cell-surface target is generally followed by an internalization. An internalized complex of a target and an imaging probe is trafficked to lysosomes, where proteins are degraded by proteolytic enzymes. Further fate of a label depends on charge, size and lipophilicity of degradation products associated with the radionuclide. If a label would provide a product that is hydrophilic and rather big (> 500 Da), it would be trapped inside the cell and be retained for a long time (Shih et al., 1994). Such labels are called *residualizing labels*. Strong residualizing properties are typical for radiometal-chelator complexes, but could also be observed when a radiohalogen is coupled to a polar or charged molecular moiety, which is non-degradable by proteolytic enzymes (Stein et al. 1995; Pruszynski et al. 2018). If radiometabolites are lipophilic, they can diffuse through lysosomal and cellular membranes and leak from cells to be further redistributed through the blood and excreted in the urine (Tolmachev et al. 2003). This type of label is called *non-residualizing*. The non-residualizing properties are typical for a majority of radiohalogen labels associated. It is important to keep in mind that the internalization rate depends on the nature of both a molecular target and an imaging probe. A rapid internalization is typical for binding of an agonistic peptide ligand to its receptor or for bivalent binding of an IgG to its antigen. Internalization of an antagonist and of monovalent binders is typically much slower and caused by renewal of cellular membrane. The use of residualizing labels is necessary to provide an acceptable retention of activity in tumours when a target-receptor complex is rapidly internalized (Reubi 2003). In the case of slow internalization, the use of residualizing label might not offer a strong advantage in the tumour uptake, especially shortly after injection (Lindbo et al. 2018b; Deyev et al. 2019).

It is important to remember that internalization takes place not only in tumours, but also in excretory organs like liver and kidneys. The kidney is the main excretory organ for protein-based molecules smaller than 60 kDa (Vegt et al. 2010). The excreted peptides and proteins are internalized by proximal tubuli cells and degraded in lysosomes to preserve amino acids inside the body. In the case of residualizing label, activity is retained in kidneys (Vegt et al. 2010). This phenomenon is typical for both antibody fragments and many scaffold proteins such as knottins, fibronectin domains, ADAPTs, DARPins and affibody molecules (Jiang et al. 2012, Garousi et al. 2015; Goldenberg et al., 1990, Hackel et al. 2012; Kramer-Marek et al. 2011 ). Although this does not complicate imaging of metastases in the lumbar region (Sörensen et al. 2014; Sörensen et al. 2016), this phenomenon contributes to an absorbed dose to patients, especially when a long-lived radiometal is used as a label. Relatively slow internalization of such ESP as affibody molecules, DARPins and ADAPTs enables the use of non-residualizing labels for imaging of tumours while maintaining low activity retention in kidneys, since renal radiometabolites clear rapidly in this case (Garousi et al. 2016a; Deyev et al. 2019; Lindbo et al. 2018b). The same phenomenon might be used for reduction of liver-associated activity with preserved tumour uptake (Vorobyeva et al. 2018; Deyev et al. 2019).

### Influence of a radiolabel on biodistribution

Apparently, radiolabelling is associated with coupling of an atom or a group of atoms to a targeting protein. This may result in modification of its biodistribution and targeting features. Conceivably, bulky mAbs are not very sensitive to such modifications and the new methods for site-specific coupling of chelators to antibodies would provide better control over composition of radiolabelled antibodies and, most likely, would improve biodistribution of antibody-based targeted probes. Still, the major effect might be expected from residualizing properties of a label. This can influence noticeably retention of activity in tumours and results in a serious difference in tumour-to-blood ratios (Brouwers et al. 2004; Malmberg et al. 2011; Steffens et al. 1998). An excessive direct radioiodination might noticeably reduce immunoreactivity of mAbs due to the high tyrosine fraction in binding sites (Nikula et al. 1995). Labelling has a little effect if a mAb is not overmodified but an excessive modification can change both biodistribution and targeting properties. For example, a coupling of up to 8 MAG3 chelators does not change the biodistribution of ^186^Re-labelled anti-CD44 antibodies (van Gog et al. 1996). An increase of chelator-to-antibody ratio from 5 to 10 lead to decrease of immunoreactive fraction of anti-CD20 antibody rituximab from 71% to 53% (Guleria et al. 2018). The effect might be quite dramatic when a lipophilic pendant group, such as an antimitotic drug auristatin E is conjugated (Lhospice et al., 2015).

In the case of small ESPs or short peptides, the modification of proteins by labelling is much more substantial, and effect on biodistribution is more pronounced. Labelling can modify binding to blood proteins, off-target interaction with normal tissues and even predominant excretion pathways (Tolmachev and Orlova 2010c). Therefore, tumour-to-organ ratios can differ several-fold depending on a label. The issue is that the influence of labelling chemistry on biodistribution of ESPs is difficult to predict as it depends both on properties of a label and properties of ESPs.

## Comparison of imaging probes

Detailed optimisation of labelling chemistry could facilitate the selection of a conjugate, providing the best sensitivity and specificity for imaging. Unfortunately, affibody molecules and, to less extend, ADAPTs are the only type of scaffold proteins that are studied in sufficient detail. This is important to remember when reading the following section. It is possible that non-optimal variants of some ESPs are taken for comparison, and the potential of some imaging probes may not be completely revealed.

Another important issue of such comparison is that only literature data are compared. Very often, different tumour models were used by different groups. We did try to select studies where the same xenografts or xenografts with approximately equal target expression level were used. Still, there might be a substantial variation in e.g. subclones of cell lines. Ideally, the comparisons should be performed in the same laboratory and using the same batch of xenografted mice. Unfortunately, we can only dream about this.

### Targets

#### HER2

**Fig. 2:**
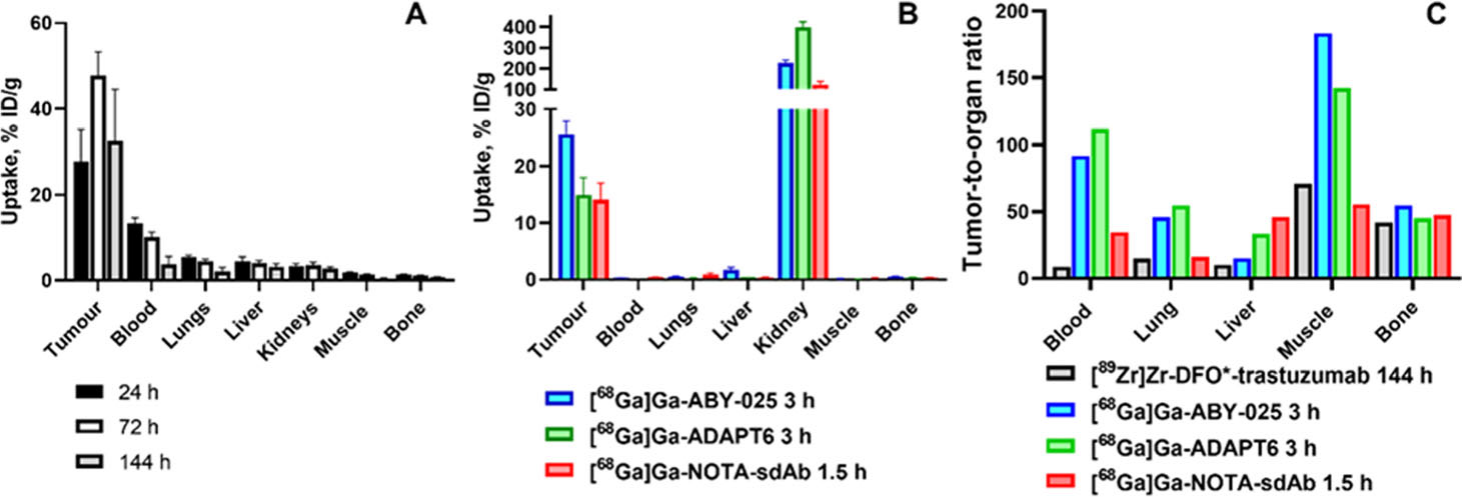
Targeting of HER2-expressing tumours in mice using positron-emitting imaging agents. **a**. Uptake of the antibody [^89^Zr]Zr-DFO*-trastuzumab in tumour, blood, kidneys and major metastatic sites. **b**. Uptake of small targeting probes in tumour, blood, kidneys and major metastatic sites. Data are from (Vugts et al. 2017). **c** Tumor-to-tissue ratios for an antibody [^89^Zr]Zr-DFO*-trastuzumab 144 h after injection (Vugts et al. 2017), [^68^Ga]Ga-ABY-025 affibody molecule 3 h after injection (Kramer-Marek et al. 2011), ADAPT [^68^Ga]Ga-ADAPT6 3 h after injection (Lindbo et al. 2018a, 2018b) and [^68^Ga]Ga-sdAb 2Rs15d 1.5 h after injection (Massa et al. 2016).

Human epidermal growth factor receptor 2 (HER2) is a tyrosine kinase receptor regulating cellular proliferation. Overexpression of HER2 has been detected in a large fractions of breast (Slamon et al. 1987) and gastroesophageal cancers (Van Cutsem et al. 2015). Tumor response to a number of HER2 targeting therapeutics correlates with the level of HER2 expression. Therefore, determination of HER2 expression level is strongly recommended in breast and gastroesophageal cancers (Wolff et al. 2013; Van Cutsem et al. 2015). Development of probes for imaging of HER2 was pursued for many years, and a number of excellent reviews have been recently published on this topic (Gebhart et al. 2016; Henry et al. 2018; Massicano et al. 2018). Therefore, we will limit ourselves here to comparison of preclinical data for representative examples of imaging probes for PET. [^89^Zr]Zr-DFO*-trastuzumab (Vugts et al. 2017) is highly likely the best antibody-based probe for imaging of HER2. It is based on the high-affinity therapeutic antibody trastuzumab and the radionuclide is stably conjugated. Its biodistribution is characteristic for a radiolabelled antibody (Fig. 2a): slow clearance from blood and normal tissues, tumour uptake peaking 72 h after injection and the highest tumour-to-organ ratios 144 h after injection.

**Fig. 3:**
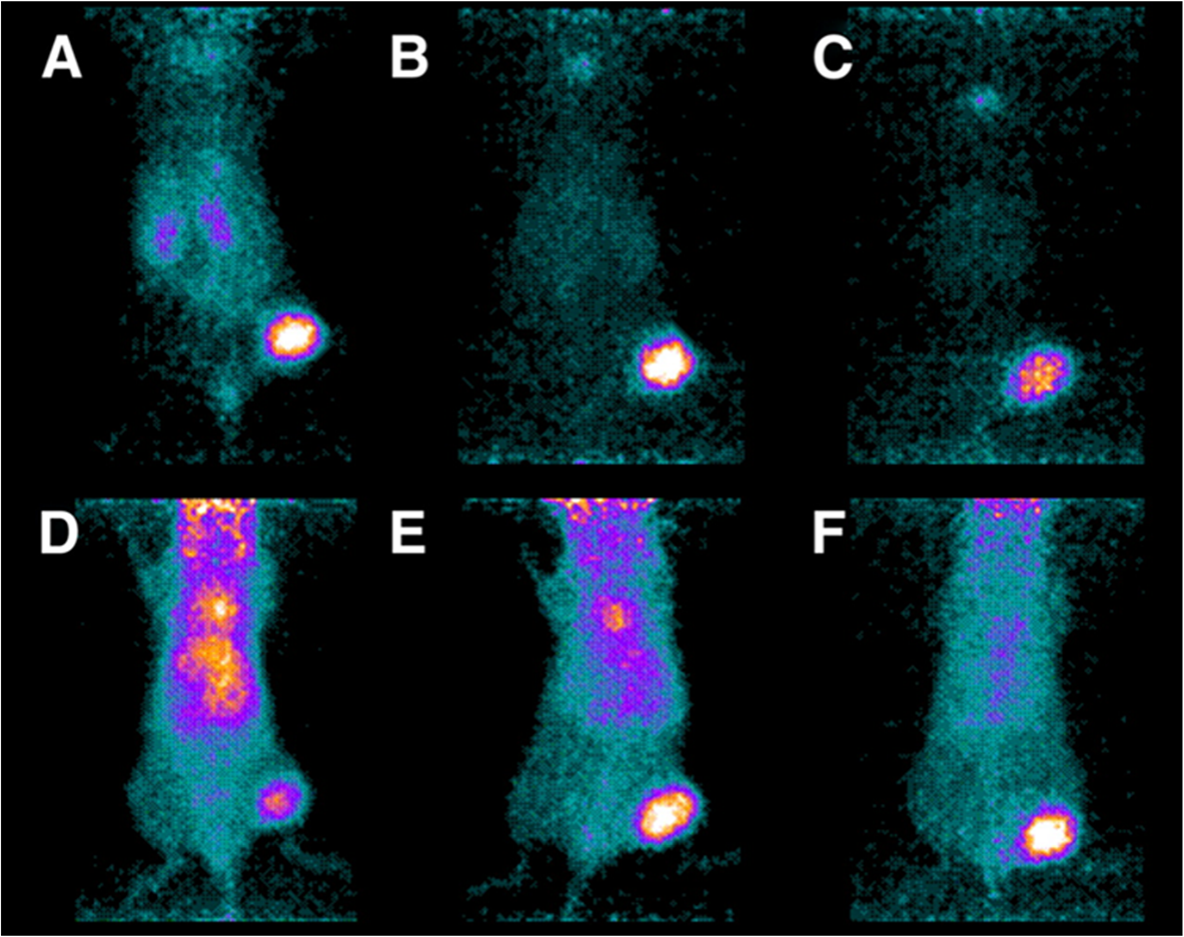
Small-animal PET images of uptake in NCI-N87 xenografts relative to other tissues of [^124^I]I-PIB-ZHER2:342 (**a**–**c**) and [^124^I]I-PIB-trastuzumab (**d**–**f**) in representative mice sacrificed at 6 (**a** and **d**), 24 (B and E), and 72 h (**c** and **f**) after intravenous injection of Affibody molecule (1.2 MBq) or of mAb (0.8 MBq). The image is reproduced from (Orlova et al. 2009)

The biodistribution pattern of sdAb and scaffold proteins labelled with ^68^Ga (Kramer-Marek et al. 2011; Massa et al. 2016; Lindbo et al. 2018a) was completely different (Fig. 2b). The highest tumour uptake was noticeably lower compared to [^89^Zr]Zr-DFO*-trastuzumab (12-26 %ID/g vs 48 %ID/g). However, the activity was cleared from a majority of tissues (except from kidneys) already by 1.5-3 h after injection. At these time points, small imaging probes provided much higher tumour-to-organ ratios compared to [^89^Zr]Zr-DFO*-trastuzumab even six days after injection (Fig. 2c). These data were in agreement with the data for the non-residualizing label. Direct head-to-head comparison of anti-HER2 ZHER2:342 affibody molecule and trastuzumab (labelled with ^124^I using the same technique) demonstrated tumour-to-blood ratio for [^124^I]-PIB-ZHER2:342 six hours after injection that was six-fold higher than for [^124^I]-PIB-trastuzumab 72 h after injection. This resulted in appreciably better imaging contrast (Fig. 3).

#### EGFR

**Fig. 4:**
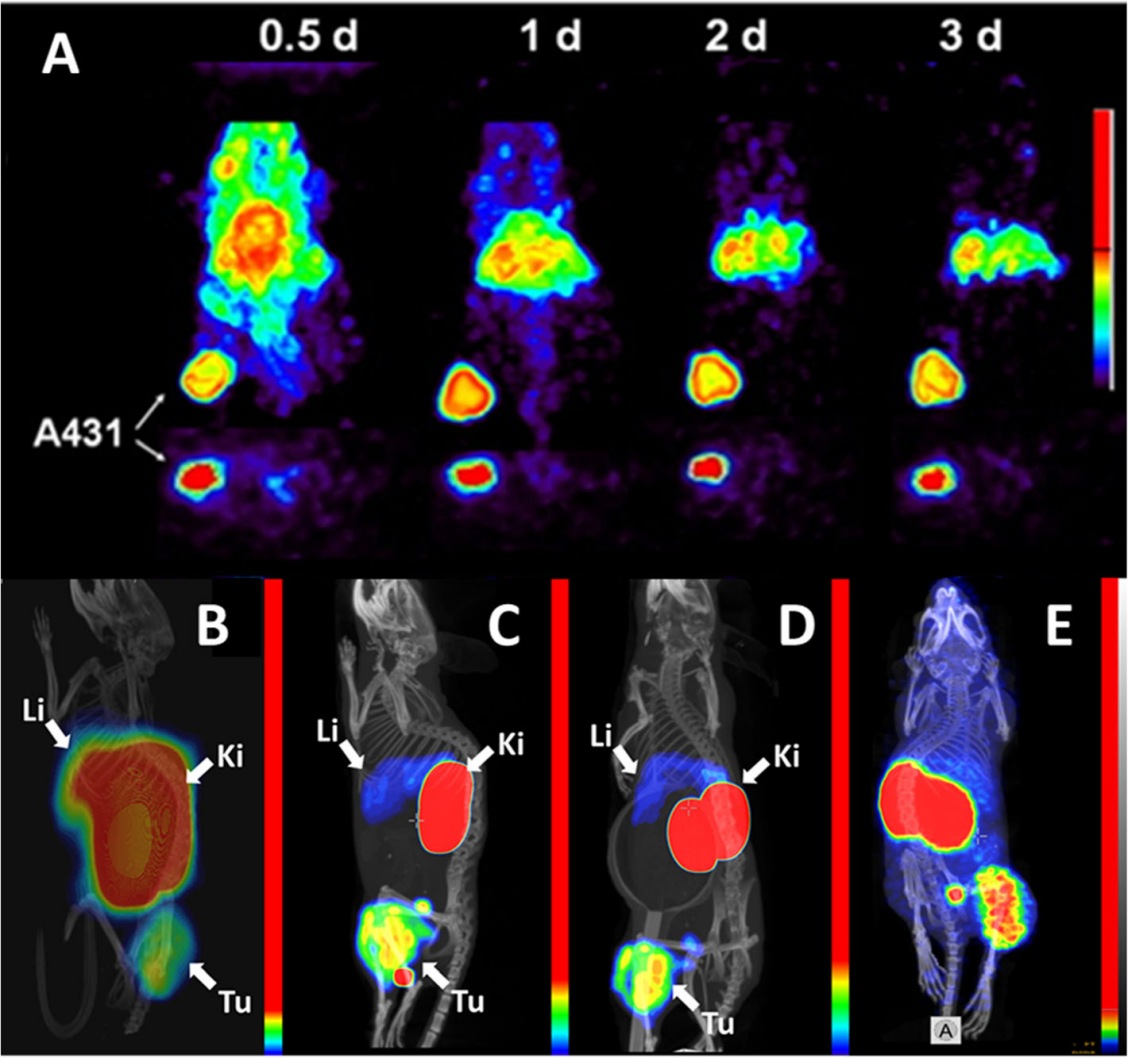
Imaging of EGFR-expression in A431 xenografted mice using antibodies and affibody molecules. Imaging using A. [^86^Y]Y-CHX-A”-DTPA-panitumumab at different time points (Nayak et al. 2010). B. [^68^Ga]Ga-DOTA-ZEGFR:2377 3 h after injection ( Garousi et al. 2017b); C. [^57^Co]Co- DOTA-ZEGFR:2377 3 h after injection; D. [^57^Co]Co- DOTA-ZEGFR:2377 24 h after injection ( Garousi et al. 2017b); E. [^68^Ga]Ga-DFO-ZEGFR:2377 3 h after injection (Oroujeni et al. 2018a, 2018b)

Epidermal growth factor receptor (EGFR) is receptor tyrosine kinase, which is overexpressed in many malignant tumours (Roskoski Jr. 2014). Overexpression of EGFR is a predictive biomarker for response/sensitivity to different therapies in non-small cell lung cancer and head and neck squamous cell carcinomas (Cappuzzo et al. 2005; Hirsch et al. 2006; Pirker et al. 2012; Bentzen et al. 2005). Therefore, several groups dedicated their research to finding optimal probes for radionuclide imaging of EGFR (Pereira et al. 2018). Therapeutic anti-EGFR antibodies, chimeric cetuximab and human panitumumab were labelled with single photon- and positron-emitting nuclides (Aerts et al. 2009; Nayak et al. 2010; Huhtala et al. 2010). One of the challenges in imaging of EGFR in tumours is a noticeable expression of EGFR in hepatocytes, which creates a natural barrier for imaging probes circulating in blood flow (Divgi et al. 1991). Clinical trials demonstrated that an increase of the injected dose of non-labelled antibody permits penetration of this barrier (Divgi et al. 1991). However, therapeutic antibodies have typically low cross-reactivity (if any) to murine EGFR, which limits the value of murine models in their pre-clinical development. It was possible to visualize EGFR-expressing xenografts using radiolabelled antibodies (see for example Table 1 and Fig. 4). Still, there was a mismatch of activity uptake in tumours and the EGFR expression level (Aerts et al. 2009; Nayak et al. 2010). Preclinical evaluation of sdAb labelled with technetium-99m for imaging of EGFR expression demonstrated that rapid blood clearance allows obtaining high contrast images shortly after administration (1-3 h pi). An absence of EPR effect permitted to discriminate between tumors with high and low EGFR expression (Huang et al. 2008; Gainkam et al. 2008; Gainkam et al. 2011). Further EGFR-targeting sdAb was labelled with gallium-68 and zirconium-89 to use for PET (Vosjan et al. 2011). However, anti-EGFR sdAbs do not bind to murine EGFR, and these studies did not address interaction with EGFR expression in normal tissues. To overcome the limitation of a mouse model, the anti-EGFR affibody molecule ZEGFR:2377 having equal affinity to human and mouse models was developed (Tolmachev et al. 2010a). It has been demonstrated that it is possible to find an amount of injected protein, which could saturate EGFR in liver without saturating receptors in tumours (Tolmachev et al. 2010a). Further studies demonstrated that the interaction of radiolabelled affibody molecules with EGFR on hepatocytes is not the only mechanism of hepatic uptake. The physicochemical properties of a chelator-radionuclide combination has also a strong influence on hepatic uptake. Labelling of ZEGFR:2377 with ^111^In and ^68^Ga using maleimido derivative of DOTA conjugated to C-terminus (Tolmachev et al. 2010a; Garousi et al. 2017b) resulted in hepatic uptake exceeding tumour uptake (Table 1 and Fig. 4b). The use of the same chelator for labelling with ^57^Co (a long-lived surrogate for positron-emitting nuclide ^55^Co) resulted in the tumour uptake exceeding the hepatic uptake (Table 1 and Fig. 4c and d) (Garousi et al. 2017b). Labelling of the same affibody molecule with ^68^Ga using DFO provided (Oroujeni et al. 2018a, 2018b) also higher uptake in tumour than in liver (Table 1 and Fig. 4e). Overall, affibody molecules provided higher tumour-to-organ ratios in A431 xenograft compared to antibody-based imaging probes.

**Table 1:** Targeting of EGFR-expressing A431 xenografts in mice using antibodies and affibody molecules

Tracer	Time (h)	Tumor uptake (%ID/g)	Tumor-to-blood ratio	Tumor-to-liver ratio	Tumor-to-bone ratio	Reference
[^89^Zr]Zr-DFO- cetuximab	96	~ 4	~ 1.5	~ 0.3		Aerts et al. 2009
[^86^Y]Y-CHX-A”-DTPA-panitumumab	72	23±3	~ 3	~ 2	6-7	Nayak et al. 2010
[^57^Co]Co- DOTA-ZEGFR:2377	3	5.8±2.4	12±2	3.1±0.5	24±5	Garousi et al. 2017b
[^57^Co]Co- DOTA-ZEGFR:2377	24	4.04±0.03	32±7	3.3±0.3	21±5	Garousi et al. 2017b
[^68^Ga]Ga-DOTA-ZEGFR:2377	3	2.7 ± 0.1	7±2	0.44±0.03	12±2	Garousi et al. 2017b
[^68^Ga]Ga-DFO-ZEGFR:2377	3	8.6±2.4	6.7±2.9	2.2±0.8	16±5	Oroujeni et al. 2018a, 2018b

#### CAIX

**Fig. 5:**
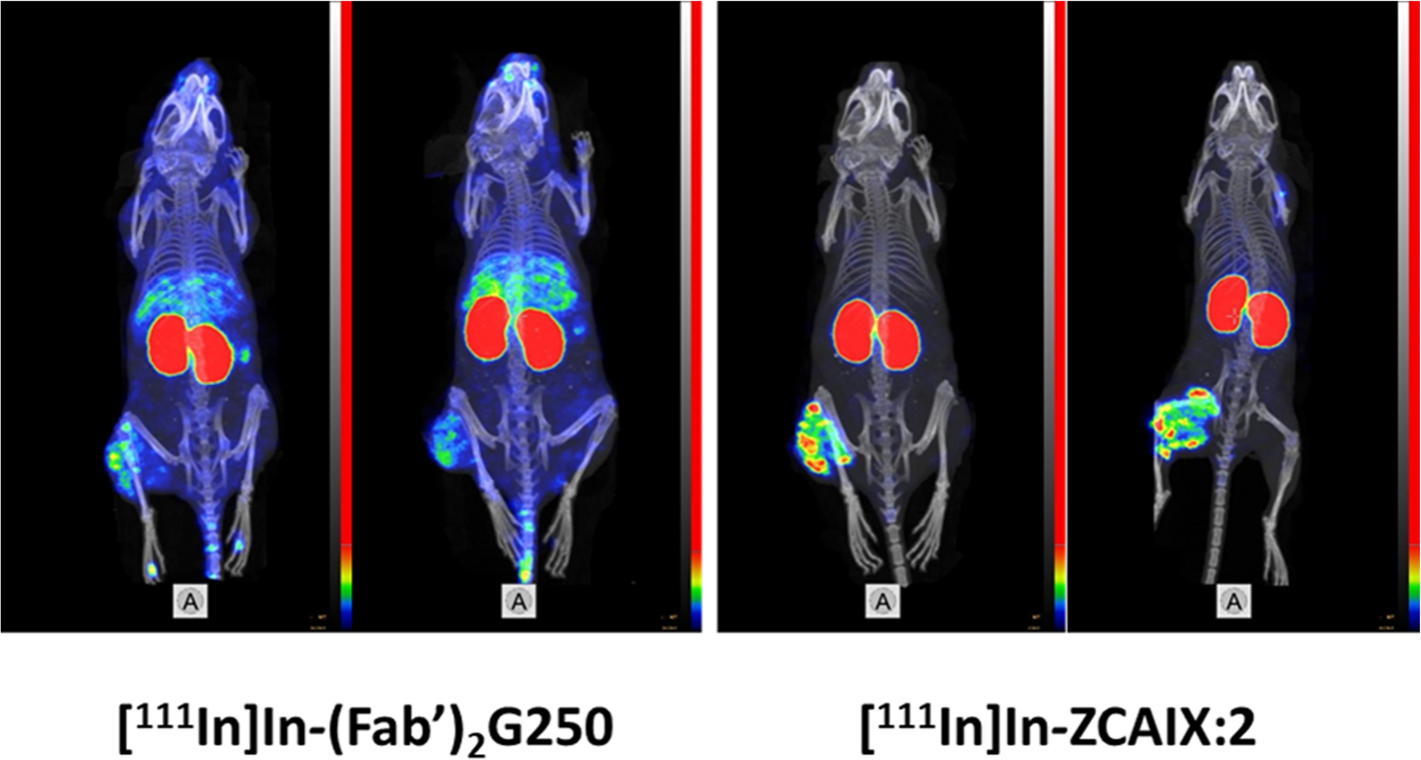
SPECT/CT imaging (4 h after injection) of CAIX-expression in SK-RC-52 xenografted mice using [^111^In]In-DOTA-ZCAIX:2 and [^111^In]In-DTPA-G250(Fab’)_2_. Images are presented as maximum intensity projection (MIP). Images are reproduced from (Garousi et al. 2019)

CAIX is a membrane-bound cell-surface enzyme, which is overexpressed in a large fraction of renal cell carcinomas (Krieg et al. 2000; Wiesener et al. 2001). In addition, expression of CAIX is increasing under hypoxic conditions and an elevated level of CAIX is a marker of chronic hypoxia in tumours (Potter and Harris 2004). CAIX expression significantly correlates with a higher risk of locoregional failure, disease progression, and higher risk of developing metastases (van Kuijk et al. 2016). Therefore, CAIX is considered as an important therapeutic and imaging target (Supuran 2008; Tafreshi et al. 2014; Wichert and Krall 2015). Thus, development of radiolabelled anti-CAIX for imaging and therapy was pursued by the scientific community (Stillebroer et al. 2007). Table 2 shows a comparison of preclinical literature data concerning targeting of SK-RC-52 renal cell carcinoma xenografts with a high CAIX-expression level using radiometal-labelled imaging probes. [^88^Y]Y-DOTA-G250 antibody (Brouwers et al. 2004) demonstrated an excellent tumour uptake, 44±15 and 55±11%ID/g 72 and 168 h, respectively. Still, tumour-to-organ ratios 72 h after injection were moderate, and tumour-to-blood ratio was moderate even at a later time point. In the case of [^111^In]In-DTPA-G250(Fab’)_2_ (Garousi et al. 2019b), the clearance of activity from blood was appreciably faster, and tumour-to-organ ratio for this agent 24 h after injection was ten-fold higher than for the antibody 168 h after injection. However, tumour-to-bone and tumour-to-muscle ratios were higher for the antibody-based agent. The renal uptake of activity was also very high, which is typical for tracers undergoing renal excretion and re-absorption in kidneys. [^111^In]In-DOTA-HE_3_-ZCAIX:2 provided at 4 h after injection a tumour uptake that was higher than the uptake of [^111^In]In-DTPA-G250(Fab’)_2_ but lower than the uptake of [^88^Y]Y-DOTA-G250 antibody (Garousi et al. 2019b). The renal re-absorption of activity was the highest in the case of [^111^In]In-DOTA-HE_3_-ZCAIX:2 affibody molecule. However, the activity was cleared from the majority of normal tissues providing the superior tumour-to-tissue ratios for the affibody molecule already 4 h after injection. This has been confirmed by small-animal SPECT/CT imaging (Fig. 5).

**Table 2:** Biodistribution of radiometal labelled CAIX-imaging probes in mice bearing SK-RC-52 xenografts. Results are presented as %ID/g (the mean values and standard deviation for four mice). Data for [^88^Y]Y-DOTA-G250 antibody are taken from (Brouwers et al. 2004) and tumour-to-organ ratios are calculated using uptake values. Data for [^111^In]In-DTPA-G250(Fab’)_2_ and [^111^In]In-DOTA-HE_3_-ZCAIX:2 are taken from (Garousi et al. 2019)

	[^88^Y]Y-DOTA-G250 antibody		[^111^In]In-DTPA-G250(Fab’)_2_		[^111^In]In-DOTA-HE_3_-ZCAIX:2
	72 h	168 h	4 h	24 h	4 h
Uptake, %ID/g					
Tumor	44±15	55±11	6±1	5±1	15±3
Blood	12.5±1.3	8.2±1.9	2.9±0.5	0.07±0.02	0.24±0.03
Lung			2.3±0.2	0.7±0.3	0.49±0.09
Liver	7.0±0.7	3.7±0.4	10±2	8±2	0.5±0.1
Kidney			216±30	145±9	392±26
Muscle	1.4±0.2	0.7±0.2	0.8±0.1	0.4±0.1	0.14±0.02
Bone	1.0±0.2	0.4±0.2	2.1±0.4	1.4±0.2	0.32±0.08
Tumour-to-organ ratio					
Blood	3.6	6.7	2.1±0.2	67±12	63±11
Lung			2.7±0.4	7±1	30±3
Liver	6.3	14.9	0.7±0.3	0.6±0.2	33±2
Kidney			0.029±0.005	0.03±0.01	0.038±0.008
Muscle	31.7	78	7.9±0.7	11±1	102±20
Bone	44	137	3.0±0.5	4±1	47±8

## Conclusion

Radioimmunodetection evolved from experimental tool for tumour staging to the immunoPET, a powerful method for evaluation of pharmacokinetics and target engagement in development of new biotherapeutics. New formats of protein-based imaging probes, such as sdAb and engineered scaffold proteins, have emerged. Preclinical and early clinical data suggest that these novel formats would be superior to radiolabelled antibodies for stratification of patients for targeted therapies. These promising data are waiting for confirmation in prospective clinical trials.

## Data Availability

Not applicable

## Abbreviations

[^18^F]-FDG: [^18^F]-2-fluoreo-2-deoxy-D-glucose
EPR effect: Effect of enhanced permeability and retention of macromolecules in tumours
mAbs: monoclonal antibodies
SPECT: Single photon emission computed tomography
PET: Positron emission tomography
RID: Radioimmunodetection
RTK: Receptor tyrosine kinase
EGFR: Human epidermal growth factor receptor
HER2: Human epidermal growth factor receptor type 2
GMP: Good manufacturing practice
IgG: Immunoglobulin G
EpCAM: Epithelial cell adhesion molecule
CEA: Carcinoembryonic antigen
PSMA: Prostate-specific membrane antigen
MMR: Macrophage mannose receptor
PD-L1: Programmed death-ligand 1
